# Metabolic reprogramming of macrophages and its involvement in inflammatory diseases

**DOI:** 10.17179/excli2020-3053

**Published:** 2021-03-11

**Authors:** Chunyu Guo, Rayhanul Islam, Shichen Zhang, Jun Fang

**Affiliations:** 1 Department of Toxicology, School of Public Health, Anhui Medical University, and Key Laboratory of Environmental Toxicology of Anhui Higher Education Institutes, No 81 Meishan Road, Hefei 230032, China; 2 Faculty of Pharmaceutical Science, Sojo University, Ikeda 4-22-1, Kumamoto 860-0082, Japan; 3 Department of Maternal, Child and Adolescent Health, School of Public Health, Anhui Medical University, China

**Keywords:** macrophage, metabolic reprogramming, polarization, inflammation, tissue repair

## Abstract

Macrophages are critical effector cells of the innate immune system. The presence of microbes or the stimulation by inflammatory factors triggers the metabolic reprogramming of macrophages or macrophage polarization into two phenotypes: the classically activated macrophages (M1) displaying a pro-inflammatory phenotype and the alternatively activated macrophages (M2) having anti-inflammatory functions. The imbalance between the two phenotypes has been linked with various pathological states, such as fibrosis, hepatitis, colitis, and tumor progression. An avenue of potential therapeutic strategies based on macrophage polarization has emerged. Therefore, it is essential to understand the mechanisms of macrophage polarization. In this review, we focus on the macrophage polarization process and discuss the stimuli-dependent conversion into M1 and M2 phenotypes. We also present the metabolic patterns supporting their specific functions. The factors and signaling cascades involved in intra-class switching are also detailed. Finally, the role of macrophage polarization in disease progression is discussed.

## Introduction

The immune system comprises three levels of defense mechanisms: physical barriers, such as the skin and mucosa of the digestive and respiratory tracts, non-specific innate immunity carried out by phagocytic cells, and adaptive immunity. Phagocytic cells, including neutrophilic granulocytes, dendritic cells, and monocytes/macrophages, engulf foreign particles and thus constitute a non-specific defense against invaders called innate immunity (Schumann, 2016[52]). Macrophages are the main subset of phagocytic cells and play an important role in innate immune defense. Furthermore, some macrophages also function as antigen-presenting cells to effector T cells, which contribute to the third line of immune defense, namely adaptive immunity. Tissue-resident macrophages are macrophages constantly present in various tissues, such as alveolar macrophages in the lung, microglia in the brain, Langerhans cells in the epidermis, and Kupffer cells in the liver (Porta et al., 2015[47]). Circulating monocytes contribute to the homeostasis of tissue-resident macrophages by differentiating into macrophages (Bain et al., 2016[4]; Michaeloudes et al., 2020[43]; Tabas and Bornfeldt, 2016[55]). The dynamic balance between the renewal program of blood monocytes and the loss of progenitor-derived macrophages (Bain et al., 2016[4]) is associated with pathological, inflammatory, and homeostatic reactions (Varol et al., 2015[58]).

Under normal circumstances, macrophages, called “M0 macrophages” or naive macrophages, are quiescent and not polarized immune cells (Ramond et al., 2019[50]). They are metabolically non-effective and require a metabolic reprogramming to provide adenosine triphosphate (ATP) necessary to their function. The metabolic reprogramming has two potential outcomes: the “classically activated macrophages” also called pro-inflammatory macrophages or M1 macrophages and the “alternatively activated macrophages,” which are anti-inflammatory macrophages or M2 macrophages (Ramond et al., 2019[50]; Schumann, 2016[52]). Some studies use a nomenclature based on the function; M1 macrophages are analogous to T-helper cell 1 (Th1), whereas M2 macrophages are analogous to T-helper cell 2 (Th2) (Tabas and Bornfeldt, 2016[55]; Weigert et al., 2018[64]). The role of the macrophage metabolic reprogramming in the host protection from invaders and in disease progression has evolved. In this review article, we present the macrophage polarization process. We report the changes in the metabolic pattern involved in macrophage polarization and the underlying signaling pathways. Finally, we describe the role of macrophage polarization in various diseases and point out therapeutic strategies based on the interaction between macrophages and the host tissues.

## Metabolic Reprogramming of Macrophages

### Metabolic reprogramming in M1 phenotypic expression

M0 macrophages differentiate into the classic pro-inflammatory M1 phenotype in response to various stimuli such as toll-like receptor (TLR) ligand (i.e., lipopolysaccharide [LPS]) and cytokines (i.e., interferon-γ [INF-γ] and tumor necrosis factor α [TNFα]) (Olefsky and Glass, 2010[44]; Wilson et al., 2019[65]).

In M1 polarization, the tricarboxylic acid (TCA) cycle is interrupted at two checkpoints: after the citrate and the succinate generation steps. However, the detailed mechanism is not completely known (O'Neill and Pearce, 2016[45]). As a result, citrate and succinate accumulate in the cell (O'Neill and Pearce, 2016[45]), and cellular energetics are disturbed. Succinate is an intermediate of the electron transport chain (ETC) in the aerobic respiration and promotes the production of reactive oxygen species (ROS). It also stabilizes the level of hypoxia-inducible factor-1α (HIF-1α), a key signaling molecule for M1 class switching. The role of HIF-1α is discussed extensively below in this manuscript (Liu et al., 2017[40]; Van den Bossche et al., 2017[57]).

Additionally, pro-inflammatory macrophages shift to aerobic glycolysis to meet up the cellular energy demand (Verdeguer and Aouadi, 2017[59]). This process is known as the “Warburg effect” and refers to the metabolic reprogramming of cells where ATP is generated solely by glycolysis while the TCA cycle is inactive even in the presence of oxygen (Kelly and O'Neill, 2015[32]; O'Neill and Pearce, 2016[45]; Wilson et al., 2019[65]). In aerobic glycolysis, each molecule of glucose yields only two ATP molecules and produces lactate as an end product. However, it is a rapid energy production process and supports the cell during acute inflammation at the early stages of infection (Chowdhury et al., 2019[10]; Ramond et al., 2019[50]).

**Figure 1:**
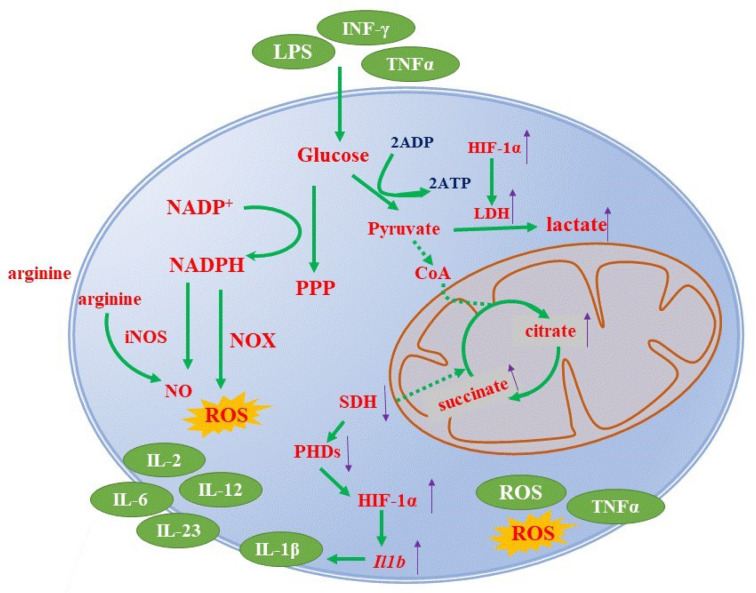
Metabolic reprogramming into M1 phenotype. Stimulation with LPS, INF-γ, or TNFα of macrophages favors a reprogramming toward the pro-inflammatory phenotype. One of the very first events is the TCA cycle halt resulting in the accumulation of citrate and succinate. The dysfunction of the TCA cycle activates a metabolic process known as the “Warburg effect.” The accumulation of succinate inhibits the succinate dehydrogenase (SDH) increasing the level of HIF-1α, a signaling molecule characteristic of the M1 phenotype. Inducible NO synthase, adenosine 5′-monophosphate-activated protein kinase (AMPK) pathway, ROS, and other pathways are also involved in this metabolic system. See text for details.

The crucial function of classically activated M1 macrophages is to defend against acute deleterious pathogens by releasing pro-inflammatory cytokines (such as interleukin-12 [IL-12], IL-1β, IL-2, IL-6, IL-23, and TNF-α), nitric oxide (NO), ROS, and chemokines. The accumulated succinate serves as basic material to produce inflammatory cytokines. Figure 1(Fig. 1) summarizes the signaling pathways involved in metabolic reprogramming into M1 phenotype.

### Metabolic reprogramming in M2 phenotypic expression

The activation of the alternative M2 anti-inflammatory phenotype is triggered by the stimulation with IL-4, IL-13, IL-10, transforming growth factor-β (TGF-β), and glucocorticoids (GCs), all of which inhibit the function of pro-inflammatory macrophages.

**Figure 2:**
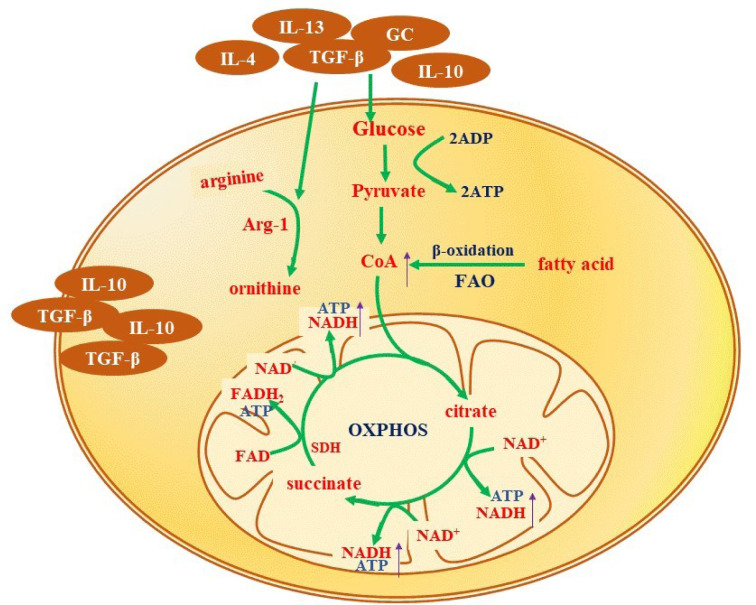
Metabolic reprogramming in M2 phenotype. The stimulation with IL-4, IL-13, IL-10, TGF-β, or GC provokes the metabolic reprogramming of macrophages into the anti-inflammatory phenotype (M2). In M2 macrophages, arginine is metabolized to collagen and ornithine by arginase-1 (Arg-1) in the urea cycle. The M2 phenotype relies on FAO and OXPHOS to generate energy. FAO supplies energy to generate acetyl-CoA which enters the TCA cycle and OXPHOS producing higher amounts of ATP than glycolysis. Besides, the anti-inflammatory macrophages can yield pro-resolving molecules, such as IL-10 and TGF-β, thus promoting tissue repair and remodeling. See text for details.

In contrast to the pro-inflammatory subset, anti-inflammatory macrophages rely on fatty acid oxidation (FAO) and oxidative phosphorylation (OXPHOS). The integrity of the TCA cycle and oxidative metabolism is maintained in M2 macrophages (Liu et al., 2017[40]; Van den Bossche et al., 2017[57]). The OXPHOS is supported by FAO and glucose in anti-inflammatory macrophages. The expression of CD36, a lipid scavenger receptor, is also increased in M2 macrophages (Figure 2(Fig. 2)) (Wilson et al., 2019[65]). FAO supplies energy to generate acetyl-coenzyme (CoA), which further enters the TCA cycle (Chowdhury et al., 2019[10]) to generate ATP via OXPHOS. OXPHOS produces higher amounts of ATP (30-32 from one glucose molecule) compared with glycolysis. Figure 2(Fig. 2) summarizes the signaling pathways involved in metabolic reprogramming into M2 phenotype.

M2 macrophages produce pro-resolving molecules, such as IL-10 and TGF-β. They contribute to tissue repair and remodeling via promoting the resolution of inflammation. They also provide a defense against parasitic infections (Genard et al., 2017[18]; Tabas and Bornfeldt, 2016[55]; Tacke and Zimmermann, 2014[56]; Verdeguer and Aouadi, 2017[59]).

## Molecular Pathways Involved in the Macrophage Metabolic Reprogramming

### Arginine metabolic pathway

In macrophages, the expression of inducible NO synthase (iNOS) is increased after stimulation with either LPS or INF-γ or both, both of which promoting the polarization of macrophages to the pro-inflammatory M1 phenotype (Figure 1(Fig. 1)) (Kelly and O'Neill, 2015[32]). In M1 macrophages, LPS induces the accumulation of citrate as a result of the TCA cycle halt. To compensate the cellular energy shortage due to the TCA cycle disruption, nicotinamide adenine dinucleotide phosphate (NADPH) is produced through the pentose phosphate pathway (O'Neill and Pearce, 2016[45]). In the presence of NADPH, iNOS metabolizes arginine imported from extracellular fluid into NO and citrulline (Baig et al., 2015[3]). Citrulline is recycled into arginine by argininosuccinate synthase 1 and argininosuccinate lyase and released in the extracellular matrix (ECM), thus sustaining the production of NO (Kelly and O'Neill, 2015[32]). NO is a reactive nitrogen species that nitrosylates iron-sulfur proteins from the ETC complexes, such as cytochrome-C oxidase, and consequently inhibits mitochondrial respiration (Kelly and O'Neill, 2015[32]). NO also exhibits antimicrobial effects at high concentrations. In addition, iNOS-derived NO plays an important regulatory role by enhancing nuclear factor-κB (NF-κB) transcriptional activity (Baig et al., 2015[3]). Simultaneously, ROS are produced in phagosomes by NADPH oxidase (NOX) (Figure 1(Fig. 1)) (Kelly and O'Neill, 2015[32]).

In contrast, arginine is metabolized to collagen and ornithine through the urea cycle enzyme arginase-1 (Arg-1) in M2 macrophages (Otoupalova et al., 2020[46]). This process decreases the level of arginine and consequently limits NO production (Figure 2(Fig. 2)) (O'Neill and Pearce, 2016[45]; Van den Bossche et al., 2017[57]). 

### HIF pathway

HIFs are heterodimeric transcription factors activated by hypoxia. HIFs are composed of α-subunits (HIF-1α, HIF-2α, and HIF-3α) and a β-subunit (HIF-1β) (Gonzalez et al., 2018[20]; Susen et al., 2019[54]). They are highly conserved members of Per-Arnt-Sim, which is a subfamily of the helix-loop-helix family (Corcoran and O'Neill, 2016[11]; Gonzalez et al., 2018[20]; Susen et al., 2019[54]).

Under normoxic conditions, the α-subunits of HIFs are hydroxylated by prolyl hydroxylases (PHDs) and then ubiquitinated by E3 ubiquitin ligase. As a result, the half-life of HIF-1α is very short (less than 5 min), whereas HIF-1β is expressed continuously (Corcoran and O'Neill, 2016[11]; Susen et al., 2019[54]). The stability of HIF-*α *subunits plays an essential role in macrophage polarization.

HIF-1α is mainly involved in M1 polarization, while HIF-2α influences the polarization toward the M2 phenotype (Choe et al., 2014[9]). Additionally, succinate dehydrogenase (SDH) inhibition results in the accumulation of succinate and increases the bioavailability of HIF-1α by inhibiting PHDs. As a consequence, the transcription of target genes, such as IL-1β, is increased (Corcoran and O'Neill, 2016[11]; O'Neill and Pearce, 2016[45]). Under normoxic conditions, HIF-1α enhances glycolysis by translocating into the nucleus and binding to target hypoxia response genes, such as the glycolytic enzymes: glucose transporter-1 (GLUT1), hexokinase-2, and fructose-2, 6-bisphophatase (Kelly and O'Neill, 2015[32]; O'Neill and Pearce, 2016[45]). Moreover, lactate dehydrogenase expression is increased in the presence of HIF-1α. As a result, the formation of lactate from pyruvate is increased, which prevents the degradation of pyruvate into acetyl-CoA by the glycolysis pathway (Kelly and O'Neill, 2015[32]). Additionally, HIF-1α increases pyruvate dehydrogenase kinase (PDHK) expression. PDHK inhibits pyruvate dehydrogenase, which catalyzes the production of acetyl-CoA from pyruvate (Kelly and O'Neill, 2015[32]; Wang et al., 2019[62]). HIF-1α also directly regulates several pivotal glycolytic enzymes. Finally, HIF-1α is also known to impair anti-inflammatory responses by inhibiting the signal transducer and activator of transcription (STAT) 3 signal (Hayek et al., 2019[25]). Altogether, these data indicate that HIF-1α switches the metabolic reprogramming of macrophages toward the M1 phenotype and promotes pro-inflammatory response (Figure 1(Fig. 1) and 2(Fig. 2)). Metallothioneins (MTs) are divalent cation binding proteins. The expression levels of MTs are enhanced by stress or inflammatory stimuli, such as IL-4 and IL-13. MTs seem to downregulate the activation of HIF-1α and might therefore suppress pro-inflammatory response (Chowdhury et al., 2019[10]).

HIF-2α shares the same hypoxia response element as HIF-1α and therefore targets the same genes, including GLUT1. However, each subunit also has specific unique target genes (Keith et al., 2011[31]). HIF-2α accumulates in response to IL-6, IL-13, and IL-14 and favor the anti-inflammatory phenotype of macrophages, as evidenced by *Arg-1* gene transcription activation (Porta et al., 2015[47]). A previous study has shown that HIF-2α increased the expression of Arg-1 by activating peroxisome proliferation activator receptor-γ (PPAR-γ), which contributes to the metabolic reprogramming toward the M2 phenotype (Wang et al., 2018[61]). HIF-2α could thus modulate the polarization of macrophages by controlling the balance between Arg-1 and iNOS expression levels (Gonzalez et al., 2018[20]).

### Adenosine 5′-monophosphate-activated protein kinase (AMPK) pathway

AMPK directs macrophage polarization toward the M2 phenotype (Qiu et al., 2019[49]). AMPK is a heterotrimeric serine-threonine kinase activated by adenosine and is a vital factor for OXPHOS regulation (Qiu et al., 2019[49]; Wang et al., 2019[62]). AMPK induces catabolic pathways. For example, it upregulates carnitine palmitoyltransferase 1α (Kelly and O'Neill, 2015[32]). In the mitochondria, AMPK facilitates the uptake of fatty acids for β-oxidation. It also induces the expression of OXPHOS-related proteins, such as SDH and peroxisome proliferator activated receptor-γ coactivator-1β (PGC1β) (Kelly and O'Neill, 2015[32]). SDH hydrolyzes succinate, resulting in the decrease of succinate, which inhibits M1 polarization. Additionally, AMPK regulates nicotinamide phosphoribosyl transferase (NAMPT). NAMPT is the rate-limiting enzyme in the NAD+ salvage pathway and is associated with the activation of anti-inflammatory signaling (Audrito et al., 2015[2]; Gruenbacher et al., 2019[22]). IL-10 is an essential effector molecule of this process. In anti-inflammatory macrophages, NAD+ also stimulates the production of IL-8 (Gruenbacher et al., 2019[22]).

### Influence of ROS

ROS, such as superoxide (O_2_^-^) and hydrogen peroxide (H_2_O_2_), potentially kill microbes in macrophages and therefore play a critical role in host defense (Cheresh et al., 2013[7]). H_2_O_2_ activates the NF-κB pathway by enhancing the DNA-binding capacity of p65 NF-κB. It also stimulates p65 NF-κB phosphorylation, which increases the expression of the heterodimer NF-κB (p50-p65) (Chiang et al., 2017[8], Genard et al., 2017[18]). NF-κB (p50-p65) promotes pro-inflammatory gene transcription resulting in M1 polarization (Genard et al., 2017[18]).

NOX contributes to the production of constitutive ROS (i.e., O_2_^−^) in macrophages (Brune et al., 2013[6]; Griffiths et al., 2017[21]). O_2_^−^ from phagosomes is disputed to H_2_O_2_ that diffuses into the cytoplasm. O_2_^−^ activates the Akt and mitogen-activated protein kinase (MAPK) pathways (Griffiths et al., 2017[21]). Akt/PKB signaling activates the mTOR pathway. MAPK signaling stimulates the NF-κB pathway, which subsequently leads to the activation of HIF-1α and glycolysis (Griffiths et al., 2017[21]). Furthermore, ROS suppresses IL-1β production by promoting the hydroxylation of HIF-1α by PHDs (Early et al., 2018[15]). However, brain and muscle ARNT-like 1 (BMAL1), a core orchestrator of the molecular clock, was reported to promote the transcription of nuclear factor erythroid 2-related factor 2 (Nrf2) during this progress. Nrf2 inhibits IL-1β production by regulating directly the transcription of IL-1β or by inducing an antioxidant response to inhibit ROS (Early et al., 2018[15]).

## Macrophage Intra-Class Switching and its Regulation

The polarization of macrophage into pro- or anti-inflammatory phenotypes is mostly studied *in vitro*. Therefore, the *in vivo* processes are not completely clear. It was suggested that macrophages initially polarize into the M1 pro-inflammatory phenotype in response to infection. Then, the phenotype switches to anti-inflammatory M2 to inhibit the pro-inflammatory response and promote the tissue repair process (Liu et al., 2017[40]; Otoupalova et al., 2020[46]).

Furthermore, the functions of macrophages change dramatically during transient inflammation, including their role in cellular debris clearance, pathogen killing, stimulation of the adaptive immunity, and tissue repair (Tabas and Bornfeldt, 2016[55]). Thus, a certain functional flexibility is maintained in the polarization of pro- or anti-inflammatory macrophages, and it is difficult to draw a clear line between M1 and M2 phenotypes based on their function. The current knowledge of macrophage intra-class switching is summarized below.

### M2 to M1 polarization

**Figure 3:**
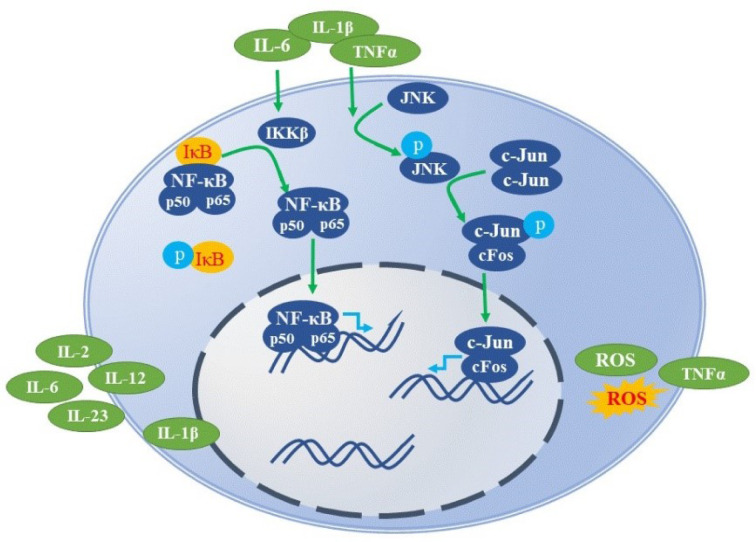
Signaling pathways triggering pro-inflammatory (M1) reprogramming. Stimulation by IL-6, IL-1β, and TNFα triggers IKKβ to phosphorylate and degrade IκB, resulting in the release of NF-κB (p50-p65). NF-κB (p50-p65) enters the nucleus and binds to its target DNA response elements, activating pro-inflammatory genes. In addition, JNK is phosphorylated and activates c-Jun, subsequently activating pro-inflammatory target genes, which are partly common with NF-κB. See text for details.

Macrophages secrete various factors, such as IL-6, IL-1β, and TNFα that activate inflammatory pathways, including NF-κB inhibitor kinase (IKKβ)/NF-κB and Jun N-terminal kinase 1/activator protein 1 (JNK1/AP1) signaling pathways (Eguchi et al., 2013[16]). Under normoxic condition, NF-κB forms a complex with the NF-κB inhibitor (IκB) and is localized in the cytoplasm (Olefsky and Glass, 2010[44]). The above-mentioned secreted factors activate IKKβ inhibitors, which phosphorylates IκB (Wilson et al., 2019[65]). IκB is then degraded, and NF-κB (p50-p65) is released. NF-κB (p50-p65) translocates to the nucleus and binds to its target DNA response elements, stimulating the expression of pro-inflammatory genes (Figure 3(Fig. 3)) (Genard et al., 2017[18]; Olefsky and Glass, 2010[44]; Zhong et al., 2017[67]).

The polarization into a pro-inflammatory macrophage also requires JNK (Han et al., 2013[24]). JNK belongs to the MAPK superfamily and is a key modulator of many cellular events, including cell proliferation and programming (Qian et al., 2018[48]). The factors secreted by macrophages stimulate the phosphorylation of JNK, which in turn phosphorylates the N-terminus of c-Jun (Olefsky and Glass, 2010[44]). Afterward, c-Jun dimers are replaced by c-Jun/cFos heterodimers, which activate the same pro-inflammatory target genes as that of NF-κB (Olefsky and Glass, 2010[44]). Increased JNK activation by the TAK1/ MKK7/JNK signaling pathway promotes a phenotypic switch of macrophages from an anti-inflammatory to a pro-inflammatory state (Figure 3(Fig. 3)) (Guo et al., 2019[23]).

### M1 to M2 polarization

**Figure 4:**
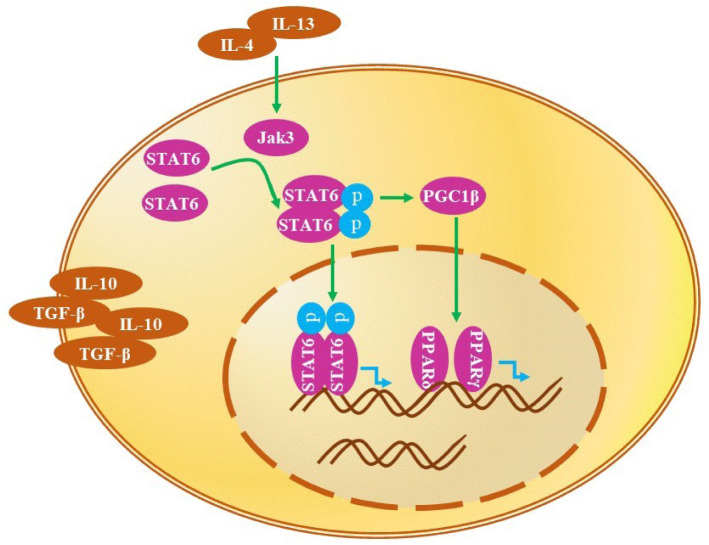
Signaling pathways triggering anti-inflammatory (M2) reprogramming. IL-4 and IL-13 induce the tyrosine phosphorylation of STAT6, leading to the formation of phosphorylated STAT6 dimers. These enter the nucleus and bind to the target DNA, enhancing the expression of anti-inflammatory genes. In addition, the phosphorylated STAT6 dimers use PGC1β as a coactivator to promote the anti-inflammatory metabolic reprogramming of macrophages by PPARγ and PPARδ. See text for details.

The macrophages are maintained in an anti-inflammatory state by STAT6 and PPARs, which are activated by IL-4 and IL-13 (Eguchi et al., 2013[16]; Jensen et al., 2011[28]). STAT6 is required for anti-inflammatory macrophage polarization (Czimmerer et al., 2018[12]). STAT6 phosphorylation by Janus-tyrosine kinase 2 /STAT3 is upregulated by erythropoietin, thus promoting anti-inflammatory polarization (Wang et al., 2017[63]). IL-4 activates STAT6 axis, whereas IL-6 stimulates STAT3 axis (Spence et al., 2013[53]). IL-4 and IL-13 induce the tyrosine phosphorylation of STAT6 resulting in the formation of phosphorylated STAT6 dimers. These dimers use PGC1β as a coactivator to promote the stimulation of the anti-inflammatory metabolic reprogramming of macrophages by PPARγ and PPARδ (Kang et al., 2008[30]; Olefsky and Glass, 2010[44]). Indeed, PPARγ activates many inflammation-associated transcription factors, including AP1, NF-κB, and STATs, leading to the inhibition of the pro-inflammatory response (Lefevre et al., 2015[37]). PPARδ regulates the oxidative metabolism at the level of transcription and improves insulin sensitivity, which is considered to be involved in the reprogramming (Figure 4(Fig. 4)) (Kang et al., 2008[30]).

## Involvement of Macrophage Polarization in Inflammatory Disorders

Dysfunctional macrophage phenotypes might contribute to disease development. Dysregulated pro-inflammatory macrophages were reported to cause collateral tissue damage, which contribute to metabolic diseases (atherosclerosis and diabetes) and autoimmune diseases, such as multiple sclerosis and arthritis (Ju and Mandrekar, 2015[29]).

Dysregulation of anti-inflammatory macrophages was involved in chronic diseases, such as tissue fibrosis, asthma, and atopic dermatitis (Ju and Mandrekar, 2015[29]). Therefore, controlling the balance between pro- and anti-inflammatory macrophages might be a valuable strategy to treat the related diseases.

### Tumor

Tumor-associated macrophages (TAMs) are important components of the tumor microenvironment. TAMs play an important role in modulating tumor growth, progression, invasion, and metastasis. At the early stages of cancer, TAMs behave like M1 macrophages displaying anti-tumor immune functions. However, it gradually switches to the M2 phenotype, which facilitates tumor progression by suppressing anti-tumor immunity (Allavena et al., 2008[1]). Additionally, M2 TAMs mainly promote the tumor growth by stimulating angiogenesis and accelerating tissue remodeling (Dong et al., 2017[14]). Tumor vascularization is stimulated by HIF-1α and HIF-2α, which modulate the production of VEGF (Liu et al., 2019[39]). M1-like TAMs, just like tumors, are supported by the “Warburg effect” and utilize glycolysis for generating ATP and lactate even in the presence of oxygen. Lactate and hypoxia generated by the “Warburg effect” can increase the expression of *Vegf* and *Arg-1*. The latter promotes the shift of the pro-inflammatory to the anti-inflammatory phenotype (Liu et al., 2019[39]). In this context, reversing the macrophage polarization toward the M1 phenotype from hypoxia-induced M2 macrophages has been considered as a potential strategy for cancer treatment. This novel therapeutic approach was exploited by inducing M2 to M1 class switching through the NF-κB pathway modulation (Chiang et al., 2017[8]; Ryan et al., 2015[51]).

### Pulmonary fibrosis 

Pulmonary fibrosis is characterized by the accumulation of ECM in alveolar cells, which results in architectural remodeling, impaired gas exchange, and breathing difficulty (Mahalanobish et al., 2020[42]). Pulmonary fibrosis is caused by repeated and continuous micro-damage and subsequently, abnormal repair of alveolar epithelial cells (Hosseinzadeh et al., 2018[27]). The alveolar resident macrophages participate in both these aspects of the pathogenesis of pulmonary fibrosis as they are involved in the clearance of dead cells by phagocytosis and in tissue restoration. Macrophages exert either a pro- or anti-fibrotic role depending on the stage of fibrosis progression. Anti-inflammatory macrophages ensure the pro-fibrotic function by: 1) inducing the recruitment and proliferation of fibroblasts; 2) producing tissue inhibitors of metalloproteinase (TIMPs); 3) inhibiting the degradation of ECM; and 4) promoting epithelial to mesenchymal transition (EMT) (He et al., 2019[26]; Kolahian et al., 2016[33]; Wang et al., 2020[60]). In summary, all the pro-fibrotic roles of M2 macrophages are associated with abnormal tissue repair. The main anti-inflammatory regulatory factors are TGF-β, IL-1β, and platelet-derived growth factor (Goda et al., 2020[19]). Cumulative evidence suggests that anti-inflammatory macrophages play a major role in the progression of pulmonary fibrosis. Thus, inhibiting polarization into the M2 anti-inflammatory phenotype might provide potential treatment options for pulmonary fibrosis.

The polarization of macrophages is also associated with oxidative stress, which is a critical event during pulmonary fibrosis. ROS are generated from the mitochondria in alveolar macrophages. Mitochondrial dysfunction enhances the generation of H_2_O_2_ and O_2_^−^, which are then released into the cytosol from the mitochondrial ETC (Cheresh et al., 2013[7]; Liu and Chen, 2017[41]). O_2_^−^ is further converted to H_2_O_2_ by superoxide dismutase. The generated ROS promote the polarization of macrophages from the pro- to the anti-inflammatory phenotype (Kurundkar and Thannickal, 2016[35]). A recent study found that inhibiting the differentiation of anti-inflammatory macrophages suppresses EMT and consequently attenuates pulmonary fibrosis (Wang et al., 2020[60]).

### Pulmonary fungal infection

In case of an infection by microbial pathogens, such as fungi in the lungs, macrophages are the first responders. However, the state of the macrophage polarization determines the progression or resolution of the disease. Pro-inflammatory macrophages stimulated by IFN-γ ensure fungicidal function as they promote the generation of microbicidal factors such as NO (Davis et al., 2013[13]). In contrast, stimulation of IL-4 triggers macrophage polarizing toward the anti-inflammatory phenotype, which facilitates the deposition of collagen, fibroblast accumulation, and tissue remodeling (Leopold Wager and Wormley, 2014[38]). The anti-inflammatory effect is achieved by the upregulation of Arg-1, which metabolizes arginine into ornithine without generating microbicidal NO (Davis et al., 2013[13]; Leopold Wager and Wormley, 2014[38]). Therefore, downregulation of the anti-inflammatory phenotype or its conversion to the pro-inflammatory phenotype might constitute an effective therapeutic strategy against pulmonary fungal infections.

### Viral hepatitis

Macrophages are essential for maintaining liver tissue homeostasis. Next to the tissue-resident Kupffer cells, monocyte-derived macrophages are present in the liver (Tacke and Zimmermann, 2014[56]). Kupffer cells fight against microbial infections and promote the restoration of injured tissue. An involvement of Kupffer cells in hepatitis and fibrosis has also been reported (Krenkel and Tacke, 2017[34]). The monocyte-derived macrophages accumulate temporarily during liver injury (Krenkel and Tacke, 2017[34]). A previous study found that liver fibrosis related to the hepatitis-B virus (HBV) is associated with the accumulation of anti-inflammatory macrophages (Bility et al., 2014[5]). Anti-inflammatory macrophages impair Th1 immune response after HBV infection, whereas pro-inflammatory macrophages promote Th1 immune response to clear the virus particles (Bility et al., 2014[5]).

Hepatitis C virus (HCV) infection leads to the dysfunction of inflammatory processes that lead to a polarization halt thereby impairing both pro- and anti-inflammatory differentiation (Fan et al., 2015[17]). However, in chronic infection, HCV antigen induces M2 polarization and simultaneously suppresses M1 polarization by inhibiting the expression of NF-κB binding protein (Fan et al., 2015[17]). Zhang et al. reported that HCV impedes the polarization of monocyte-derived macrophages through the TLR2 signaling pathway (Zhang et al., 2016[66]).

A recent contradictory study showed that HCV infection could activate STAT3 and impede the activation of STAT1 (Kwon et al., 2019[36]). Altogether, HCV might impair both the pro- (M1) and anti-inflammatory (M2) polarization but is mostly involved in the loss of the pro-inflammatory phenotype.

## Conclusion

The macrophage metabolic reprogramming or class switching from M1 to M2 and vice-versa plays a crucial role in disease progression and recovery. In inflammatory injuries, M0 macrophages are classically activated into M1 macrophages, which display pro-inflammatory, antibacterial, or anti-tumor functions at the early stages. Over time and under various stimuli, the M1 subset is polarized to alternatively activated M2 macrophages, which are anti-inflammatory in nature and show a protective role against parasitic attack by supporting tissue repair and remodeling. The antibacterial and anti-tumor immune responses from the M1 macrophages are executed by releasing pro-inflammatory cytokines (such as IL-12, IL-1β, IL-2, IL-6, IL-23, and TNF-α), NO, ROS, and chemokines. In contrast, M2 macrophages produce pro-resolving molecules, such as IL-10 and TGF-β, to ensure their antiparasitic and anti-inflammatory role. Table 1(Tab. 1) summarizes the classification of M1/M2 macrophages and signal pathways involved in their polarization. Taken together, both M1 and M2 phenotypes have protective functions.

However, M1 macrophages may damage tissues by producing an excessive amount of bactericidal cytokines. M2 macrophages have been reported as potential contributors to various diseases, such as fibrosis and cancer. M2 macrophages promote tumor progression by supporting angiogenesis and tissue remodeling. During the pathogenesis of tissue fibrosis, anti-inflammatory macrophages play a pro-fibrotic role attributed to EMT induced by the recruitment and proliferation of fibroblasts, TIMPs production, and ECM potentiation.

Therapeutic strategies based on the current knowledge of this class switching phenomenon already showed promises regarding the understanding of the pathogenesis and disease management particularly concerning fibrosis, hepatitis, colitis, and cancer. More detailed studies of macrophage class switching mechanisms in diseases might provide insightful clues for devising effective therapeutics in the future.

## Notes

Shichen Zhang and Jun Fang (Faculty of Pharmaceutical Science, Sojo University, Ikeda 4-22-1, Kumamoto 860-0082, Japan; Tel: +81-96-326-4137, Fax: +81-96-326-5048, E-mail: fangjun@ph.sojo-u.ac.jp) contributed equally as corresponding authors.

## Conflict of interest

The authors declare no conflict of interest.

## Figures and Tables

**Table 1:**
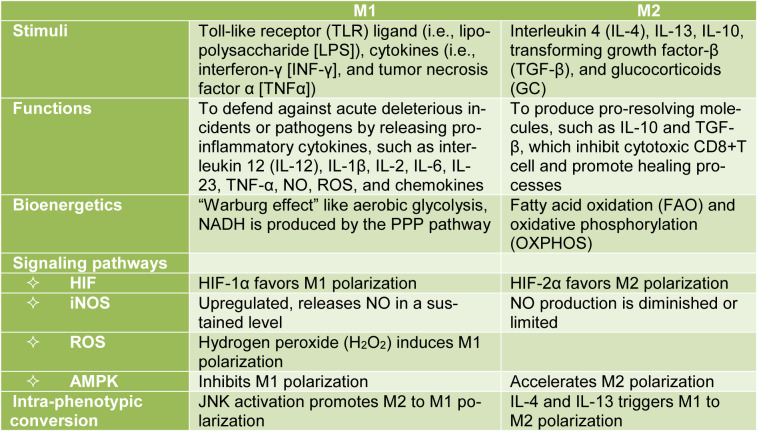
Classification of M1/M2 macrophages and signaling pathways involved in their polarization
